# Patients with chronic pain: evaluating depression and their quality of life in a single center study in Greece

**DOI:** 10.1186/s40359-019-0366-0

**Published:** 2019-12-21

**Authors:** Ekaterini Rapti, Dimitrios Damigos, Paraskevi Apostolara, Vasiliki Roka, Chara Tzavara, Christos Lionis

**Affiliations:** 1 Medical Center of Ag. Theodoroi-Loutrakiou, Dimitriou Stamou 8 St, Ag. Theodoroi, Corinthia, Greece; 2 Medical Psychology, Laboratory, Medical Psychology, University of Ioannina, Medical School, University of Ioannina, Ioannina, Greece; 3 Nursing Department, University of West Attica, Scientific Partner of Faculty of NursingNational and Kapodistrian University of Athens, Athens, Greece; 4 Hellenic Navy, Navy Hospital of Athens, Scientific Partner of Faculty of Nursing, Adjunct Academic Staff, MSc Health Care Management, National and Kapodistrian University of Athens, Hellenic Open University, Athens, Greece; 5 Biostatistician, Centre for Health Services Research, Department of Hygiene, Epidemiology and Medical Statistics, Medical School, University of Athens, Athens, Greece; 6 General Practice and Primary Health Care, Clinic of Social and Family Medicine, Medical School, University of Crete, Heraklion, Greece

**Keywords:** Chronic pain, Depression, Quality of life, BPI, PHQ-9, Euro- 5 D

## Abstract

**Background:**

Chronic pain constitutes one of the most common reasons for seeking health care services and may even lead to disability. Chronic pain has been associated with depression and deterioration of the quality of life. The aim of our study is to outline the burden of chronic pain in the context of a primary health care (PHC) setting in Greece and to investigate its association with depression and quality of life.

**Methods:**

A cross-sectional study was conducted from September 2016 to November 2016. The subjects of the study comprised 200 individuals who visited the regional medical center of Ag. Theodoroi, Greece. The collected data were from a representative sample of 200 adults and included demographic data, social and medical history, presence and characteristics of chronic pain and questions from three questionnaires for the assessment of pain (BPI- short form), the investigation of depression (PHQ-9) and the evaluation of the quality of life (EuroQ-5D) validated in Greek language. Multiple regression analysis was used in order to find associated factors with quality of life, depression and chronic pain.

**Results:**

A percentage of 56.8% of the participants, the majority of whom (62%) were women, reported chronic pain. Among individuals with pain, lower back area was the most common location. Based on the given questionnaire, depression was detected in 22. 5% of the participants who claimed chronic pain. Regression analyses revealed that women and respondents with chronic mental disorders like depression and anxiety had significantly higher scores on the pain scale and suffered pain which had a greater impact on their daily activities. According to regression analysis decreased quality of life was expressed by women, as well as participants with a chronic mental disorder. A significant reverse correlation emerged between the quality of life, depression and pain scales.

**Conclusion:**

Chronic pain, as it has been studied within this PHC setting, is a common health care problem. Individuals who had experienced chronic pain and depression had a lower health-related quality of life.

## Background

Pain is an unpleasant experience, with biological, cultural, religious and philosophical aspects [1]. Chronic pain (CP) constitutes one of the most common reasons for seeking care in primary health care [2].

It is estimated that one in five Europeans suffers from chronic pain (CP); thus, significant burden is transferred to individuals, families and careers, as well as to healthcare providers and national economies [3]. Studies performed in different settings have demonstrated that CP affects between 10 and 30% of the adult population in Europe [3]. The American Academy of Pain Management has characterized pain as the “silent epidemy”, while in the USA, more than 50 million Americans suffer from chronic pain due to illness, disability or accident [4]. Also, insufficient pain management constitutes a major problem for sufferers which could be avoided with the application of a well-organized pain management program [5]. Worldwide, several Ministries of Health, the World Health Organization (WHO) and the International Association for the Study of Pain (IASP) highlight the necessity of an interactive approach to pain management and suggest a multidimensional response in terms of care [3].

While CP is known to be relatively common, prevalence is estimated to be highly variable. As referred to in the National Health Interview Survey in 2016, the prevalence of CP among U. S adults was 20.4 and 8.0% of them had high-impact chronic pain [6]. A systematic review of epidemiological studies of CP (5) reported a range of estimates (from 2 to 40%) across 15 studies. They suggested that this variability might be due to differences in the population and the mode of data collection [7]. According to a recent study, which was conducted in the UK, the estimated prevalence of chronic pain was 43% [8]. At the same time the prevalence of patients with chronic pain among the general population in Spain was 16.6%, with at least one person affected in every four Spanish homes [9]. Consequently, chronic pain affects people variously and constitutes a medical problem and not just a symptom.

People with chronic pain also have a significantly higher prevalence of depression compared to those who do not report chronic pain [10]. Many chronic pain patients have typical disabilities: they frequently experience depression, anxiety, sleep disturbance, fatigue [11], and they experience a deterioration in the quality of life (QoL) [12].

However, although chronic pain may be considered as a prognostic factor for depression, while depression constitutes a prognostic factor for chronic pain [13, 14], this relation should receive greater attention in primary care research. People seek medical help, not only to relieve the pain but also because pain affects social and work function and their daily activities and causes emotional discomfort [15, 16]. It also has a negative impact on family relations, social interaction and professional life. As a result, they experience a significant deterioration in the quality of life and consequently depression and disability [17].

The majority of patients with chronic pain are usually seen in primary health care (PHC) setting in most countries [2]. Studies conducted on these patients in PHC are very significant as such studies might help to improve the identification and management of these patients. Unfortunately, both mental health disorders and pain have not received the attention that deserved in PHC in Greece. In this country, PHC reform is underway, a focus on both clinical entities has been considered as an interesting and challenging idea.

For the above reasons, we turned our attention to chronic pain. The main areas of study were three:

- To assess the burden of chronic pain among the people who visited the primary care center (PCC) in a specific area of Greece
- To identify pain characteristics like duration, frequency and location
- To examine the impact of chronic pain on depression and quality of life

## Methods

The study sample was a random of individuals who visited the PCC for any reason. For the purpose of the study, chronic pain is defined as constant pain or pain that flares up frequently, and has been experienced for at least 3 months [18].

The sample consisted of 200 individuals who were assessed using questionnaires. One of the main strengths of this study is that used questionnaires were with many parameters such as socioeconomic, demographic and health characteristics of participants. This helped to collect data that allows estimating better the study population, allows to describe more association between chronic pain and these characteristics, can be used several times and it may sufficiently characterize patient’s pain.

### Setting

The present cross-sectional study was conducted in one PCC in a suburban region named Ag. Theodoroi, in the Prefecture of Corinthia, Greece, between September 2016 and November 2016. It is a public primary health care unit, in which two general practitioners work, and is located about 65 km south-west of Athens. The registered permanent residents are 4643. However, during summertime, due to its proximity to tourist resorts and destinations, the target group triples. The educational level of the population is relatively low, with only 30% having completed compulsory education, and where only 10% have completed higher education. Of the permanent residents, 2.945 are economically inactive, while those in employment are numbered approximately 1.500 [19].

### Participants, and sample

Participants were people who visited the medical center for any reason, they were above 18 years of age and younger than 75 years of age and they spoke Greek fluently.

**Fig. 1:**
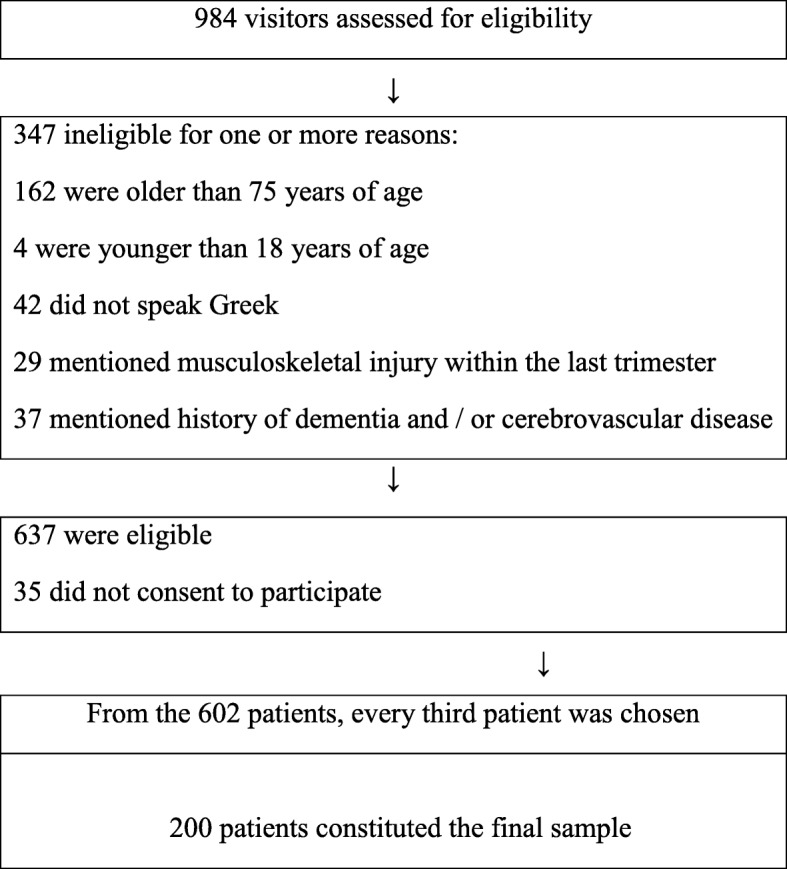
Flow Chart of the Sample

As described in the flow chart given (Fig. 1), a total of 984 individuals visited the medical center of which 637 met all the inclusion criteria. Of these, 35 refused to participate in the study. For the remaining 602, a selection was used; thus, every third patient was asked to complete a questionnaire either by himself or with the aid of the researcher. All in all, the sample of the study included 200 individuals who had visited the medical center for whatever reason. The participants that completed the questionnaire and those that refused had similar demographics concerning age and sex.

In addition to the restrictions mentioned above, were excluded:

• Those with lower limb amputation.

• Those with musculoskeletal trauma suffered in the last 3 months.

• Those that were unable to walk.

• Those with schizophrenia or other psychotic disorder.

• Those that had been diagnosed with neurological conditions such as stroke, multiple sclerosis, Parkinson’s disease and polyneuropathy.

### Data collection tools

Data was collected in printed form, including׃

*An information letter*, describing the purposes of the study, its duration and how to respond to the questionnaires*.*

*A letter of consent*, for participating in the research meant to be signed.

*A demographics form,* comprising of questions regarding the subject’s full social and medical history. We collected data about family status (married/ single/divorced/widowed), educational status (primary school/ middle or high school/ university), smoking (yes or no), alcohol consumption (one drink per day or more) and data about their medical history (chronic disease, such as cardiovascular disease, diabetes, chronic obstructive pulmonary disease and chronic mental disorder such as depression and anxiety). The form was created by the researcher for the needs of the study.

*The questionnaire for pain assessment, Brief Pain Inventory (BPI),* which includes 9 questions related to pain occurring within the last 24 h. The BPI rates the intensity of pain, as well as the interference caused by the pain. The Pain Severity rating is the average of 4 items, which are scored from 0 (no pain) to 10 (acute pain), while the Pain Interference rating is the average of 7 items (0 meaning no interference and 10 meaning complete interference); relative mood, physical activity, work, social activity, relationships, sleep and the joy of life. The BPI questionnaire has been translated into Greek and validated in a palliative care unit for cancer patients [20]. Approval for the use of the particular questionnaire has been obtained by the author [21].

*The questionnaire for the investigation of depression in Primary Health Care Services, Patient Health Questionnaire-9 (PHQ-9)*: PHQ-9 is related to 9 symptoms relevant to the 9 criteria of DSM-IV, concerning depression syndrome experienced within the last two weeks. Each of the criteria is rated from 0 (absence) to 3 (almost daily). A validation study is available in Greek [22]. For the diagnosis of depression the cutoff point 10 was used, given the fact that the questionnaire’s validation in Greek was administered to rheumatic patients, and the optimal cutoff point being 10 with sensitivity 81.2%, and specialty 86.7% [22]. This is also in line with previously published international studies since the most commonly mentioned cutoff points for depression diagnosis is 10 [23]. For the use of PHQ-9 no reproduction or translation permission is required as it is available via the internet.

*The questionnaire for quality of life assessment Euro- 5 D*: Euro Qol- 5 D-3 L shows five dimensions of the quality of life assessment: mobility, self-care, daily activities, anxiety and depression [24]. Each one is classified within three levels of severity. The EQ-5D instrument has been validated in the Greek general population [25].

### Statistical analysis

Continuous variables were expressed as mean values (SD) or with median and interquartile range (IQR), while categorical variables were expressed as absolute and relative frequencies. For the comparison of proportions, the Fisher’s exact tests were used. The normality assumption was evaluated using Kolmogorov-Smirnov test. Student’s t-tests were used for the comparison of continuous variables between two groups and analysis of variance (ANOVA) was used for the comparison of continuous variables between more than two groups. Univariate comparisons for BPI dimensions were made using Mann-Whitney and Kruskal -Wallis tests because the distribution was not normal. Pearson’s or Spearman’s correlation coefficients (r) were used to test the association of two continuous measures.

Linear regression analysis in a stepwise method (p for entry 0.05, p for removal 0.10) was used in order to find independent factors associated with BPI dimensions, EQ-5D and PHQ-9 in subjects with chronic pain. For BPI dimensions, log-transformations were made, due to their skewed distribution. Adjusted regression coefficients (β) with standard errors (SE) were computed from the results of the linear regression analyses. Also, standardized regression coefficients were performed as a measure of the effect of independent variables. Additionally, R squared was reported as the percent of the variance explained by the model. Independent variables initially entered in the model for BPI dimensions, were demographics, while independent variables for PHQ-9 were demographics and BPI dimensions. Independent variables initially entered in the model for EQ-5D, were demographics, BPI dimensions and PHQ-9, while having a chronic mental condition was not included because of its correlation with PHQ-9. The variables that entered into the models were checked for multicollinearity using tolerance value and Variance Inflation Factor (VIF). No problems of multicollinearity were diagnosed since for all independent variables that finally entered into the models the tolerance value was more than 0.2 and the VIF was less than 10. Sample size of 200 participants, was chosen in order to achieve a 95% power to detect significant differences at the 0.05 level of significance and at an effect size of 0.13 or more, via regression analysis. According to Cohen (1988) values of effect size near 0.02 are considered small, near 0.15 are considered medium and above 0.35 are considered large. Thus we choose the value of 0.13 that is near 0.15 in order to have enough power to reveal significant findings of medium or large effect sizes [26]. All reported *p* values are two-tailed. Statistical significance was set at *p* < 0.05 and analyses were conducted using SPSS statistical software (version 19.0).

## Results

### Descriptive data

The sample consists of 200 participants (76 men and 124 women) with an average age of 62.2 years (old) (SD = 14.5). Sample characteristics are presented in Table 1. One hundred and ninety five of the participants were Greek (97.5%) and 69 (34.5%) were smokers. One hundred and forty (70%) of the study-population reported a chronic disease and 34 (17.1%) a mental disorder: 23 (11.5%) of them had been diagnosed by a physician with depression, while 24 (70.6%) of the participants who had been diagnosed with a mental disorder had been using the medication.

**Table 1:** Sample characteristics

	Ν (%) Ν = 200
SOCIODEMOGRAPHIC	
Age, mean (SD)	62.2 (14.5)
Sex	
Men	76 (38.0)
Women	124 (62.0)
Family status	
Married/ in a relationship	155 (77.5)
Single/ Divorced/ Widowed	45 (22.5)
Children	
No	24 (12.4)
Yes	169 (87.6)
Nationality	
Greek	195 (97.5)
Other	5 (2.5)
Working	44 (22.0)
Educational status	
Primary school	85 (42.7)
Middle/High school	78 (39.2)
University	36 (18.1)
Smoking	69 (34.5)
Alcohol consumption	40 (20.0)
**CLINICAL**	
Chronic disease	140 (70.0)
Chronic mental disorder	34 (17.1)
Medication for mental disorder	24 (70.6)
Diagnosed by a physician with depression	23(11.5)
Chronic pain	113 (56.8)
Duration of pain (years), median (IQR)	5 (2–10)
Specialist consultation for pain relief	90 (80.4)
Pain depends on weather changes	80 (72.1)
Pain Severity Score, mean (SD)	1.78 (2.32)
Pain Interference Score, mean (SD)	1.92 (2.77)
Depression score PHQ-9, mean (SD)	4.34 (4.15)
PHQ-9 > 10	31 (16.6)
EQ-5D score, mean (SD)	0.74 (0.27)
Health status (VAS), mean (SD)	73.41 (19.78)

### Main results

#### The burden of chronic pain

One hundred and thirteen of the sample (56.8%) reported an experience of chronic pain, according to the criterion definition used for this study, with a median duration of 5 years (IQR: 2–10 years). For 105 out of the 113 patients (92.6%), the pain was located, in at least one part of the musculoskeletal system. Fifty five of these patients (48.6%) the pain was located in more than two regions, in 57 cases identified as being on the lumbar spine (50.5%), in 41 cases on lower limbs (36.3%) and in 32 cases on upper limbs (28.3%). Of those who reported chronic pain, 46 (41%) were relieved with medication and 79 (70%) had consulted an orthopedic doctor, 11 (9.7%) had consulted other health provider, while 23 (20.3%) had never mentioned anything concerning the pain either to a doctor or to any other health provider.

#### BPI scale

The pain was assessed with the use of the ΒPI scale. The score for patients suffering from chronic pain, on the “Pain Severity Score Scale” ranged from 0 to 8.75 with an average of 3.63 points (SD = 2.06), while on the “Pain Interference Score Scale” ranged from 0 to 9.29 points with the average value being 3.95 points (SD = 2. 78). In univariate analyses (Table 2), greater Pain Severity Score was found in those with chronic mental disorder and in those that pain depends on weather changes. Also, in univariate analyses, greater Pain Interference Score was found in those with chronic mental disorder, in women and in those that pain depends on weather changes.

**Table 2:** Univariate analysis results for BPI dimensions in the group of subjects that reported chronic pain (*N* = 113)

	Pain Severity Score	Value	P	Pain Interference Score	Value	P
	Median (IQR)			Median (IQR)		
Sex						
Men	2.00 (0.00 ─ 4.25)	1087^+^	0.121^+^	1.29 (0.00 ─ 6.14)	1015^+^	0.041^+^
Women	3.00 (1.75 ─ 4.75)			3.43 (0.57 ─ 6.00)		
Age, r	0.12^‡^	107^‡‡^	0.233	0.12^‡^	105^‡‡^	0.226
Family status						
Married/ in a relationship	2.75 (0.75 ─ 4.25)	706.5^+^	0.063^+^	2.51 (0.00 ─ 5.43)	599.5^+^	0.111^+^
Single/ Divorced/ Widowed	3.63 (2.00 ─ 5.00)			4.41 (2.00 ─ 7.29)		
Children						
No	3.13 (1.88 ─ 4.88)	491.5^+^	0.468^+^	4.57 (1.79 ─ 6.71)	408^+^	0.139^+^
Yes	3.00 (1.25 ─ 4.50)			2.43 (0.00 ─ 5.79)		
Working						
No	3.00 (1.50 ─ 4.75)	806.5^+^	0.200^+^	3.29 (0.29 ─ 6.14)	739^+^	0.096^+^
Yes	2.13 (0.00 ─ 3.00)			1.43 (0.00 ─ 4.43)		
Educational status						
Primary school	4.00 (2.00 ─ 4.75)	6.92^++^	0.061^++^	4.14 (0.71 ─ 6.29)	3.75^++^	0.153^++^
Middle/High school	2.00 (1.00 ─ 3.75)			2.64 (0.00 ─ 6.00)		
University	2.13 (0.00 ─ 3.00)			1.71 (0.00 ─ 4.43)		
Smoking						
No	3.00 (1.50 ─ 4.25)	1301^+^	0.591^+^	2.57 (0.00 ─ 5.86)	1267.5^+^	0.600^+^
Yes	3.00 (1.25 ─ 5.00)			3.29 (0.00 ─ 6.14)		
Alcohol						
No	3.00 (1.50 ─ 4.50)	968^+^	0.935^+^	3.29 (0.00 ─ 5.86)	872.5^+^	0.519^+^
Yes	2.25 (0.75 ─ 6.00)			1.71 (0.00 ─ 6.14)		
Chronic physical problem						
No	2.00 (1.25 ─ 3.25)	801^+^	0.067^+^	1.43 (0.00 ─ 4.07)	739^+^	0.038^+^
Yes	3.00 (1.50 ─ 4.75)			3.29 (0.29 ─ 6.29)		
Chronic mental disorder						
No	2.50 (1.00 ─ 4.25)	751^+^	0.035^+^	2.29 (0.00 ─ 5.29)	764.5^+^	0.092^+^
Yes	3.88 (2.50 ─ 5.00)			4.14 (1.14 ─ 6.43)		
Duration of pain	.10^‡^	97^‡‡^	0.325	−0.02^‡^	95^‡‡^	0.858
Specialist consultation for pain relief						
No	2.00 (0.00 ─ 3.63)	671^+^	0.074^+^	1.00 (0.00 ─ 3.79)	648^+^	0.064^+^
Yes	3.00 (1.50 ─ 4.75)			3.43 (0.14 ─ 6.14)		
Pain depends on weather changes						
No	1.75 (0.00 ─ 3.25)	846.5^+^	0.014^+^	1.14 (0.00 ─ 5.14)	872.5^+^	0.034^+^
Yes	3.00 (1.75 ─ 4.75)			3.71 (0.50 ─ 6.14)		

‡Spearman’s correlation coefficient; ‡‡number of observations used for Spearman’s correlation coefficient; ^+^Mann-Whitney test [U-value is stated]; ^++^Kruskal-Wallis test (χ^2^ value is stated with df = 2)

Results from stepwise multiple linear regression analyses in those with chronic pain, with BPI dimensions as dependent variables, are shown in Table 3. The existence of a chronic mental disorder was associated with greater Pain Severity Score, while being female was associated with greater Pain Interference Score.

**Table 3:** Results from stepwise multiple linear regression analyses with BPI dimensions as dependent variables in the group of subjects that reported chronic pain (N = 113)

	βǁ	SEǂ	Beta¶	P
*Dependent variable: Pain Severity Score*				
Chronic mental disorder	0.14	0.07	0.19	0.048
*R square of the model = 2.6%*				
*Dependent variable: Pain Interference Score*				
Sex, women vs. men	0.16	0.08	0.20	0.041
*R square of the model = 3.3%*				

**ǁ**regression coefficient; **ǂ**Standard Error; analyses were conducted on cases with chronic pain; ¶ standardized regression coefficient

#### Depression in subjects with chronic pain

Regarding depression, 22.5% of the participants were categorized as having depression, according to PHQ-9 score, in those with chronic pain and the corresponding proportion was 8.5% in those without pain (*p* = 0.009). Univariate analysis in subjects with chronic pain (Table 4) showed a positive correlation on the depression scale with the pain scales. The more pain the participants experienced, the more their daily life was affected and more symptoms of depression did they experience. Also, greater scores of depression were found in women in single/ divorced/ widowed, in those with chronic mental disorder and in those that pain depends on weather changes.

**Table 4:** Univariate analysis results for EQ-5D and PHQ-9 in the group of subjects that reported chronic pain (N = 113)

	EQ-5D score	Value (df)	P	PHQ9 score	Value (df)	P
	Mean (SD)			Mean (SD)		
Sex						
Men	0.72 (0.25)	2.84 (103) ^+^	0.005^+^	3.18 (2.74)	−3.44 (103) ^+^	0.001^+^
Women	0.57 (0.26)			7.06 (5.60)		
Age, r	−0.05^‡^	103^‡‡^	0.634	−0.10^‡^	103^‡‡^	0.321
Family status						
Married/ in a relationship	0.64 (0.26)	2.10 (103) ^+^	0.038^+^	5.21 (4.35)	−2.07 (103) ^+^	0.041^+^
Single/ Divorced/ Widowed	0.51 (0.28)			7.84 (6.12)		
Children						
No	0.54 (0.34)	0.90 (100) ^+^	0.373^+^	7.00 (5.85)	−0.86 (99) ^+^	0.394^+^
Yes	0.62 (0.26)			5.54 (5.50)		
Working						
No	0.60 (0.27)	1.46 (103) ^+^	0.148^+^	6.19 (5.65)	−1.29 (103) ^+^	0.200^+^
Yes	0.69 (0.25)			4.43 (3.44)		
Educational status						
Primary school	0.55 (0.29)	2.67 (2. 102) ^++^	0.074^++^	6.18 (5.72)	0.57 (2. 102) ^++^	0.567^++^
Middle/High school	0.67 (0.24)			6.00 (5.80)		
University	0.68 (0.23)			4.56 (4.08)		
Smoking						
No	0.61 (0.28)	0.21 (103) ^++^	0.838^+^	5.16 (4.39)	1.69 (103) ^++^	0.095^+^
Yes	0.62 (0.24)			7.08 (5.89)		
Alcohol						
No	0.61 (0.27)	0.65 (103) ^+^	0.520^+^	6.00 (5.58)	−0.61 (103) ^+^	0.546^+^
Yes	0.65 (0.27)			5.15 (4.91)		
Chronic physical problem						
No	0.64 (0.26)	−0.38 (103) ^+^	0.702^+^	5.23 (4.97)	0.57 (103) ^+^	0.569^+^
Yes	0.61 (0.27)			6.00 (5.80)		
Chronic mental disorder						
No	0.68 (0.22)	−4.81 (103) ^+^	< 0.001^+^	4.93 (4.86)	3.28 (103) ^+^	0.001^+^
Yes	0.40 (0.31)			9.09 (6.95)		
Duration of pain	−0.04^‡^	93^‡‡^	0.686	−0.12^‡^	95^‡‡^	0.25
Specialist consultation for pain relief						
No	0.71 (0.22)	−1.84 (103) ^+^	0.069^+^	4.80 (6.14)	0.92 (103) ^+^	0.361^+^
Yes	0.59 (0.28)			6.08 (5.50)		
Pain depends on weather changes						
No	0.67 (0.27)	−1.22 (102) ^+^	0.224^+^	3.71 (2.93)	2.48 (102) ^+^	0.015^+^
Yes	0.6 (0.27)			6.52 (5.43)		
Pain Severity Score, r	−0.65^‡^	104^‡‡^	< 0.001	0.44^‡^	105^‡‡^	< 0.001
Pain Interference Score, r	−0.62^‡^	102^‡‡^	< 0.001	0.50^‡^	104^‡‡^	< 0.001
Depression score PHQ-9, r	−0.63^‡^	100^‡‡^	< 0.001			

‡Pearson’s correlation coefficient; ‡‡degrees of freedom for Pearson’s correlation coefficient; ^+^Student’s t-test [t-value (DF) is stated]; ^++^ANOVA [F-value (df_1_,df_2_) are stated]

Multiple linear regression analysis of subjects with chronic pain showed that females had significantly more depression symptoms, as well as those patients with a chronic mental disorder. Also, a higher score on the pain interference scale was significantly associated with more depression symptoms (Table 5).

**Table 5:** Results from stepwise multiple linear regression analyses for depression scale and EQ-D, in the group of subjects that reported chronic pain (N = 113)

	βǁ	SEǂ	Beta¶	P
*Dependent variable: EQ-5D*				
Pain Interference Score	−0.04	0.01	−0.42	< 0.001
PHQ9 score	−0.02	0.00	−0.42	< 0.001
*R square of the model = 52%*				
*Dependent variable: Depression scale PHQ9*				
Sex, women vs. men	2.50	1.01	0.21	0.015
Chronic mental disorder	2.84	1.12	0.21	0.013
Pain Interference Score	0.81	0.16	0.43	< 0.001
*R square of the model = 32%*				

**ǁ**regression coefficient; **ǂ**Standard Error; analyses were conducted on cases with chronic pain; ¶ standardized regression coefficient

#### Quality of life in subjects with chronic pain

Regarding the quality of life, association of study variables with EQ-5D in subjects with chronic pain as assessed using univariate analyses are shown in Table 4. Lower levels on EQ-5D were found for women, for Single/ Divorced/ Widowed and those with chronic mental disorder, in additionally, increased levels on Pain Severity Score (*p* < 0.001), Pain Interference Score (p < 0.001) and depression score from PHQ-9 (p < 0.001) were associated with lower levels of health-related quality of life.

When multiple regression analysis was conducted with EQ-5D as the dependent variable in cases with chronic pain (Table 5), it was found that Pain Severity Score and the depression score were independently associated with EQ-5D levels.

## Discussion

### Main findings and discussion in the light of literature

In this study, the results confirmed the negative impact of chronic pain and depression on the patients’ quality of life. Univariate analyses showed that individuals with chronic pain and depression had lower quality of life (HRQoL). Based on regression analysis, a significant negative correlation between quality of life and pain and depression scale was found. Therefore, the more pain the participants experienced, the worse their quality of life was. Similarly, the more symptoms of depression, they had, the worse their quality of life was. The findings agree with the literature, which confirms the reciprocal nature of the depression and chronic pain relationship and poorer quality of life in these patients. Similar results were found in two very recent studies which show that the presence of depressive symptoms, affects patients’ quality of life [27, 28]. A study in patients with fibromyalgia (FM) shows that depression has a similar and additive effect in negatively influencing the physical functioning of FM patients and their HRQoL [27]. Another study which was held in Japan in patients with back pain (CLBP) proved that depression among CLBP patients was associated with higher pain scores and lower HRQoL scores, as well as lower workforce productivity and increased health care use [28].

Regarding depression, univariate analysis in patients with chronic pain has shown a positive correlation with the pain scales. Previous studies and reviews assessed the interaction between depression and chronic pain [29–33]. Bair et al., reported that morbidity of depression and anxiety with chronic musculoskeletal pain is strongly associated with more severe pain and greater pain interference with daily activities, compared to individuals with pain only [30]. Furthermore Bair conducted a literature review, which shown that the prevalence of pain in a depressed sample and the prevalence of depression in a pain sample are higher than the prevalence rates when the conditions are individually examined [31]. In a study conducted by computer- assisted telephone reviews the prevalence of chronic pain due to any cause was 22,9%. Approximately 1/3 of the group with chronic pain had comorbid depression (7,8% of the entire sample) [32]. Most of these studies, have indicated the correlation between depression and pain, particularly examining the way through in which the danger of depression increases, depending on the various aspects of pain deterioration (e.g. severity, frequency, duration and number of symptoms). Apart from this correlation, there are rising indications that depression and pain share the same neurobiology and neuroanatomical pathways [31, 34] and studies have distinctly elucidated a significant overlap in the pathophysiological process of pain and depression. Hence, the recognition of the relationship between pain and depression can assist in the treatment of pain, since similar pharmaceutical approaches are used to treat these conditions [35].

High rates of chronic pain, and the resultant heavy burden, were found. More than half (56.8%) of patients that came to the PCC for whatever reason reported chronic pain with an average duration of 5 years. In an epidemiological study in the UK with a random sample of 5036 patients the 50.4% of patients’ self-reported chronic pain [36] while Arnow et al. found that chronic pain was present in 45% of the sample [29]. What was observed was that the proportion of the patients, who reported chronic pain, was even greater than that of the patients with chronic diseases, such as cardiovascular (46%) and diabetes (15.5%), illnesses that traditionally are categorized at the higher scale of chronic diseases [37]. Given the burden of chronic pain as a presentation in primary care, and that the vast majority of patients with chronic pain are managed in primary care, physicians should have adequate evidence, training and resources to assess and manage chronic pain. Better training, combined with the assistance of current guidelines, could play an important role in improving management of chronic pain.

The vast majority of patients (92.6%) mentioned that they were in pain, in at least one part of the musculoskeletal system, most often in the lumbar spine and then, in the joints of the lower limbs, a finding that is supported by previously published findings [38, 39]. This finding explains why a large number of patients have consulted an orthopedic doctor and physioterapist. Twenty percent (20%) had not mentioned pain at al. Even though this percentage is not high, it indicates however that there are people that suffer in silence. This finding is in agreement with another study conducted in rural Greece and has shown that most people with musculoskeletal pain do not seek care from primary care services. Researchers concluded that patients consulting the PCC due to musculoskeletal pain were more likely to be experiencing mental distress and bad physical functioning [39].

An interesting finding, according to multiple regression analysis was that women had more depression symptoms and complained more about pain, compared to men. The pain had a greater effect on their daily lives denoting a worse quality of life. In epidemiological studies, women have generally reported more pain than men [40, 41]. The reasons why this gender difference appears are still not completely clear. Several explanations have been given in previous studies, which include the different experiences per gender. Women are those who most often seek health care services, and are more willing than men to refer to pain according to several epidemiological studies [42]. Also, socio-cultural differences include the role differences between the two genders, the stereotypes and the expectations of the roles [43]. Furthermore, there is some evidence today that biopsychological factors, such as the involvement of estrogen and progesterone are possible mechanisms, while the endogenous opioid system also plays a role of importance [42–44]. Cognitive and emotional factors such as stress, depression and catastrophizing (believing something to be worse than it really is), have also been referred to as factors that contribute to different reactions concerning pain between the two genders [34].

Another finding that emerged from univariate analyses of BPI is that pain scales were found to be increased in patients that reported pain depending weather changes. Previous researchers reported that weather conditions affect chronic pain [45]. The most frequently reported physical complaints associated with the weather were joint and muscle aches [45]. The results from a study in patients with fibromyalgia showed that data for pain levels, emotional measures and weather conditions were significantly associated [46]. While, the results of a cohort study in patients with hip osteoarthritis (OA) support the general opinion of OA patients that barometric pressure and relative humidity influence perceived OA symptoms [47].

#### Impact of the study

The current study comprises one first attempt to record the chronic pain in PHC setting in Greece. Although the study is bounded by several limitations, its findings may have an impact on the clinical practice and the decision making of clinicians.

Our study revealed a high proportion of people who reported chronic pain and the negative impact it had on their psychology and quality of life. Given the fact that the general practice setting is easily accessible to the patients, the need for the physicians to be properly trained is evident. Practical guidelines and recommendations on the management of chronic pain are necessary, in order to be able to improve the medical care provided. Physicians also recognize and deal with chronic pain incidents, instead of only focusing on the disease indices. Also, more research based on Primary Care is needed to inform doctor’s assessment and their management of chronic pain.

Efficient pain management includes a multidisciplinary and integrated approach the aim of which is to minimize the pain as much as possible, but also to train the patients in the achievement of wellbeing, regardless of their chronic pain. Physicians should deal with chronic pain as a serious clinical entity, by focusing on both the physical and mental symptoms. A typical psychological assessment could be the key-point in potential depression comorbidity or other psychosomatic disorders which have a negative impact on the individuals’ quality of life. This fact constitutes a basic prerequisite so that treatment options can lead to a positive health outcome.

In this context, the correlation of depression and chronic pain seems to be strong; accordingly, screening for mental disorders should be required for anyone reporting chronic pain.

Finally, this current study probably constitutes one of the very few studies of the PHC, where pain has been measured with the BPI. While it could be assumed that the BPI does not constitute the proper tool for the assessment of pain in this kind of patient, because was originally designed to assess cancer-related pain, (and is now the most commonly used cancer pain assessment instrument) [48, 49] it has been proven an easy and accurate way to measure it. This fact shows that this particular tool can be used for the measurement of chronic pain in similar populations.

#### Limitations

Nevertheless, the survey presents certain visible limitations and weaknesses. Firstly, the study data is collected only from one practice. Thus, the external validity of this study seems to be limited and under discussion. Second, the study is cross-sectional design which limits firm statements about the direction of causality between pain and psychological dysfunction (for example depression). Third, an important limitation is the absence of measurement of pain-specific constructs (other than pain interference) such as pain catastrophizing, acceptance and self-efficacy, all of which have been associated with psychological dysfunction and quality of life in individuals with chronic pain. Nevertheless, we do not have sufficient evidence that the study findings are far from the current Greek reality; as a result, the conclusions might contribute to a broader evaluation of chronic pain and its association with depression and the quality of life of an individual. Besides, a review of current published data reveals that research activity concerning chronic pain should include a qualitative approach, like interviews, in order to investigate thoroughly (relevant) patients’ beliefs and attitudes. In this context, during interviews with patients suffering from osteoarthritis of the knee, it became obvious that individuals did not necessarily consider this kind of pain as a symptom of illness [50]. According to this point of view, the qualitative approach should be included in the methodology of such research projects, in order to obtain all useful data so that conclusions are reliable.

## Conclusion

Throughout the current study it became quite obvious that the burden of chronic pain in a Greek suburban area is high and constitutes a health issue of importance. The acknowledgement of the psychological burden of chronic pain could be ameliorated with the screening of any patient suffering inexplicable pain and/or an inexplicable deterioration of an agonizing condition.

## Data Availability

The data sets used and analyzed during the current study are available from the corresponding author on reasonable request.

## Abbreviations

BPI: Brief Pain Inventory
CLBP: Chronic low back pain
CP: Chronic Pain
Euro- 5 D: Euro Qol 5 D
FB: Fibromyalgia
HRQOL: Health- Related Quality of Life
IASP: International Association for the Study of Pain
OA: Osteoarthritis
PCC: Primary Care Center
PHC: Primary Health Care
PHQ-9: Patient Health Questionnaire-9
WHO: World Health Organization
